# Learning to detect the onset of slow activity after a generalized tonic–clonic seizure

**DOI:** 10.1186/s12911-020-01308-6

**Published:** 2020-12-24

**Authors:** Carroll Vance, Yejin Kim, Guoqiang Zhang, Samden Lhatoo, Shiqiang Tao, Licong Cui, Xiaojin Li, Xiaoqian Jiang

**Affiliations:** 1 University of Houston, Houston, TX USA; 2 School of Biomedical Informatics, UT Health, 7000 Fannin St Suite 600, Houston, TX USA; 3 Department of Neurology, McGovern Medical School, UT Health, 6430 Fannin St, Houston, TX USA

**Keywords:** Electroencephalogram, Sudden death in epilepsy, Generalized tonic–clonic seizure, Onset of slow activity, Signal detection, Machine learning, Deep learning, Neural network, Convolutional neural network, Data paucity

## Abstract

**Background:**

Sudden death in epilepsy (SUDEP) is a rare disease in US, however, they account for 8–17% of deaths in people with epilepsy. This disease involves complicated physiological patterns and it is still not clear what are the physio-/bio-makers that can be used as an indicator to predict SUDEP so that care providers can intervene and treat patients in a timely manner. For this sake, UTHealth School of Biomedical Informatics (SBMI) organized a machine learning Hackathon to call for advanced solutions https://sbmi.uth.edu/hackathon/archive/sept19.htm.

**Methods:**

In recent years, deep learning has become state of the art for many domains with large amounts data. Although healthcare has accumulated a lot of data, they are often not abundant enough for subpopulation studies where deep learning could be beneficial. Taking these limitations into account, we present a framework to apply deep learning to the detection of the onset of slow activity after a generalized tonic–clonic seizure, as well as other EEG signal detection problems exhibiting data paucity.

**Results:**

We conducted ten training runs for our full method and seven model variants, statistically demonstrating the impact of each technique used in our framework with a high degree of confidence.

**Conclusions:**

Our findings point toward deep learning being a viable method for detection of the onset of slow activity provided approperiate regularization is performed.

## Background

Recent advancements deep learning have significantly improved performance for classification and detection tasks [1, 2]. However, generalization ability is still limited due to the lack of sufficient high-quality training data for many domains. This holds true for many problems in the biomedical domain where data is often limited (especially for sub-population studies), which constraints the capacity of highly powerful supervised deep learning frameworks [3]. Since deep learning is known for requiring a considerable amount of data [4], applying it to a problem such as detection of markers (onset of slow activity) to predict critical patterns in a rare disease like SUDEP is not straightforward.

## Method

Our method attempts to build a framework to apply recent advancements in deep learning [2, 5–7] to detection problems such as detection of the onset of slow activity after a generalized tonic–clonic seizure, where availability of of training data is limited. We combine a variety of preprocessing (Resampling), regularization (Anti-aliased temporal downsampling [6], Global temporal downsampling [8], Global batch-wise *z*-scoring, Kernel regularization, [9]), and optimization (Batch size [10], Loss discount factor) techniques to work around the data paucity issue. We also develop a system for real-time visualization of our models predictions to emphasize which parts of the signal contributed most to the decision https://www.youtube.com/watch?v=cDuRsh2pSRM.

### Overview

From a high level, we feed an EEG sequence *x* into our binary classification model $y = f(x)$, which estimates the probability $y \approx P(y|x)$ that the sequence contains the onset of slow activity (i.e., label). The chosen model architecture is a residual neural network [11] utilizing stacked convolution layers [12], skip connections [11], batch normalization [7], downsampling [6], and non-linear activation functions. We train our model using mini-batch stochastic gradient descent (SGD). We implemented our model using Python 3.7 and tensorflow.keras [13]. Our full source code is available on Github: https://github.com/csvance/deep-onset-detection.

### Data

The original source of the training data *D* contains variable length sequences composed of recordings from ten pairwise offsets of two adjacent EEG electrodes [14]:

$$\begin{aligned} \{fp_1-f_7, f_7-t_7, t_7-p_7, p_7-o_1, fp_2-f_8, f_8-t_8, t_8-p_8, p_8-o_2, fz-cz, cz-pz\} \in F \end{aligned}$$

The sequences were recorded from 134 different patients, each with their own variable length sequence [14]. It follows that $|D| = 134$. The EEG sampling rate $F_s$ is 200 Hz, and each timestep $t_n$ is labeled $y \in \{0, 1\}$ for the presence of slow activity [14]. We create a training set *T* derived from this set in Sequence generation. The validation dataset *V* contains $|V| = 12345$ ten second sequences sampled from 34 patients with the same EEG channels and sampling rate [14]. Each sequence is labeled $y \in \{0, 1\}$. The validation set *V* has a class imbalance for label *y*, with $|V_{pos}| = 3,219$ and $|V_{neg}| = 9,126$.

### Inputs / output format

*Inputs* Detection of the onset of slow activity requires detection within the a short time-span in order to be clinically useful. A sequence length of 10 seconds was chosen based on this requirement. It follows that the input sequence to the model contains ${\mathbf {len}}~ {\textit{seq}}_{input} = 10r = 2000$ timesteps. Each training example contains ten sequences of pairwise offsets. Considering both the sequence length and number of channels, the input to our model has the shape $({\mathbf {len}}~ {\textit{seq}}_{input}, |F|) = (2000, 10)$.

*Outputs* Our model estimates *P*(*y*|*x*) , which is a scalar value ranging between 0 and 1. Hence, the output of our model has the shape (1, )

### Preprocessing

*Sequence generation* In order to make the maximum utilization of the original training data, we first create a set $S_{pos}$ of as many positive sequences with length ${\mathbf {len}}~ {\textit{seq}}_{input} = 2000$ as possible for an individual patient, starting with $t_f = t_{onset}$, and stopping after $t_i = t_{onset}$. For memory efficiency, a stride of 5 was used during the creation of each sequence in $S_{pos}$. We then create a disjoint set $S_{neg}$ by randomly sampling at most $|S_{pos}|$ negative sequences with replacement from a uniform distribution containing every possible negative training example (sequences with $t_f < t_{onset}$) from the same patient. This process is repeated for each patient, and the final training set *T* contains the union of each $S_{pos}$ and $S_{neg}$ set.

*Resampling* Before training, 50% of generated sequences were randomly cropped relative to the first timestep, resulting in a new sequence ${\textit{seq}}_{input}'$ with the relationship ${\mathbf {len}}~ {\textit{seq}}_{input}' = u {\mathbf {len}}~ {\textit{seq}}_{input}$, where $u \in [0.9, 1.1]$ is sampled from a uniform distribution. ${\textit{seq}}_{input}'$ was then resampled to the original length ${\mathbf {len}}~ {\textit{seq}}_{input} = 2000$. While this is a commonly used image augmentation technique for object detection [15, 16], it should also be beneficial here since we are interested in augmenting the temporal relationship between frequency and phase rather than the frequency and phase itself.

### Network architecture

Other researchers have demonstrated success with residual neural network variants for detecting complicated patterns in signals [2]. Thus, we use a similar variation of *ResNet* as a starting point with pre-activation style blocks [5] as shown in Fig. 4. Through trial and error, the first few convolution layers use a $$ D = 32 $$ dimensional kernel, before increasing to 2*D* and ending with 4*D*. Increasing $$ D = 32 $$ by factors of 2 resulted in overfitting. Likewise, reducing $$ D = 32 $$ by factors of 2 resulted in underfitting. With $$ D = 32 $$, our model has $$p = $$ 165,664 trainable parameters.

*Anti-aliased temporal downsampling* We explored several different methods of temporal downsampling in our network architecture, as well as investigating recent advancements in reducing aliasing [6]. After deciding on other hyper parameters, we trained our network with an anti-aliased version of strided downsampling. We use a three point Gaussian low pass kernel with $\sigma \approx 0.79577$ during downsampling. We use the same $\sigma$ for each of the three downsampling operations to encourage the network to learn a feature representation increasingly focused on lower frequencies. Each downsampling operation divides the temporal axis of the sequence by two.

*Global temporal downsampling* Recent papers in deep learning have increasingly relied on global pooling layers to reduce the number of trainable parameters and improve generalization for a variety of problems [8, 11, 17]. We considered several different global downsampling strategies including global max pooling (GMP), global average pooling (GAP) [8], and flattening. GAP was excluded because it may not be able to effectively handle sequences where only a small percentage contains the onset. Flattening significantly increases the number of trainable parameters, and may bias towards certain parts of the sequence in the training set. GMP provides the largest activation value from each channel regardless of where it occurred. With these considerations in mind, GMP was selected for global temporal downsampling on the top of the network.

### Online augmentation

During training, online augmentations were employed to help the network to learn how to handle differences in variance and bias from patient to patient. We employ global batch-wise *z*-scoring, when combined with a small stride size during sequence generation, smaller batch sizes, and sample-wise shuffling results in the network being forced to generalize to a considerable number of different scales and biases.

*Global batch-wise z-scoring* *z*-scoring was done along batch, temporal, and channel axes, normalizing the entire batch using a single mean and standard deviation. Let $B_n$ be a mini batch of shape $(|B|, \mathbf{len }~{\textit{seq}}_{input}, |F|) = (16, 2000, 10)$ for a batch size of 16. Each mini batch $B_n$ is randomly sampled without replacement from a uniform distribution during the start of every training epoch. We calculate the mean $\mu _{batch}$ and standard deviation $\sigma _{batch}$ by reducing all three axes to a single scalar value. We then apply standard *z*-scoring as follows $B_{train}' = \frac{B_{train} - \mu _{batch}}{\sigma _{batch}}$. $B_{train}'$ is then used to calculate the loss during training. When validating our models performance, we instead *z*-score the validation set using the training set population mean and standard deviation.

### Loss

Since our neural network is a binary classifier, we used a binary cross-entropy based cost function to train the network.

*Kernel regularization* In order to encourage the model to not overemphasize a small subset of learned features which may be biased towards the training set, we used $L_2$ kernel regularization. $\lambda = 0.01$ was chosen for the $L_2$ penalty for all convolution kernels using through trial and error [9].

*Loss discount factor* While GMP may help with cases where only a small part of the onset is present, some positive sequences generated using our methodology only contain a small number of positive time steps which may negatively impact convergence. If more data was available, we could simply omit ambiguous regions during training. Due to data paucity however, another solution is needed. We define a cost discounting function $\alpha (p)$ where *p* is defined as the number of positive time steps in a sequence divided by the total length of the sequence:

$$\begin{aligned} \alpha \left( p = \frac{n_{pos}}{\mathbf{len }~seq_{input}}\right) = {\left\{ \begin{array}{ll} 0.95 &{} p = 0 \\ 10p &{} 0< p \le 0.1 \\ 1 &{} 0.1 < p \\ \end{array}\right. } \end{aligned}$$

This effectively discounts loss during the first second after the onset, starting from complete discount at $t_{onset} = t_{final}$ and ending with no discount at $t_{onset} = t_{final} - r$, with our discount linearly decreasing as $t_{onset} \rightarrow t_{final} - r$. Since our classes are balanced, we chose to discount a proportional amount from all negative examples in order to avoid bias. Finally, we define our cost function as:

$$\begin{aligned} loss(y_{true}, y_{pred}) = \alpha \cdot bce(y_{true}, y_{pred}) + \lambda \sum _{i=1}^p \beta ^2 \end{aligned}$$

### Optimization

We optimized our network during training using mini-batch stochastic gradient descent (SGD).

*Batch size* We used a mini-batch size of 16 during each training step. While a much higher batch size could easily fit into memory during training, smaller batch sizes result in a wider range of scale and bias when utilizing batch-wise *z*-scoring. Smaller batch sizes have also been observed to have a regularizing effect on the model when training with SGD [10].

*Training parameters* We selected an initial learning rate of $\eta _{i} = 0.0001$, decaying by a factor of 2 every 15 epochs for a total of 75 epochs. Momentum was set to $\beta = 0.9$.

*Experimental setup* While developing our method, we observed a high variability of outcome with different random seeds. In order to test the reliability of our methods, we conducted ten runs using different random seeds with our method during training.

*Method variants* In addition to our full method, we applied the same experiment setup to different variants omitting batch-wise *z*-scoring, $L_2$ kernel regularization, and anti-aliased downsampling. For the *z*-scoring variant, we normalize each sequence with its own mean and standard deviation during training and validation. The $L_2$ variant simply omits the $L_2$ penalty. The method without anti-aliased down-sampling performs a strided down-sampling before the residual connection, and adds a max pooling layer on the residual in order to match the sequence lengths. Two additional variants use batch sizes of 32 and 64. Finally, we created a baseline variant without batch *z*-scoring, $L_2$ regularization, anti-aliased downsampling, and the discount factor. For this variant we selected to use a batch size of 64. All variants share the same ten random seeds used in the full method for comparison.

*Metrics* Due to class imbalance in the validation set, we use receiver operator characteristic area under curve (ROC-AUC) to evaluate the accuracy of our model. Despite the imbalance, are also interested in the trade off between sensitivity and specificity for each of our variants. To compute sensitivity and specificity, values of $y_{pred} > 0.5$ are considered true, and values of $y_{pred} \le 0.5$ are considered false. The same threshold also applies for accuracy.

## Results

Accuracy over ten training runs is shown in Table 1. Table 2 shows the best single validation ROC-AUC of each variant. Finally, Table 3 shows the result of 20 additional training runs for our full method.

**Table 1:** Comparing our full method to methods which omit one technique: ten runs $\mu \pm \sigma$

Method varriant	ROC–AUC	Sensitivity	Specificity	Accuracy
Baseline	$0.600 \pm 0.024$	$0.446 \pm 0.081$	$0.689 \pm 0.057$	$0.626 \pm 0.026$
Batch size = 64	$0.646 \pm 0.027$	$0.468 \pm 0.064$	$0.732 \pm 0.042$	$0.664 \pm 0.022$
W/o $L_2$	$0.651 \pm 0.018$	$0.486 \pm 0.064$	$0.747 \pm 0.049$	$0.679 \pm 0.028$
W/o batch *z*-score	$0.658 \pm 0.034$	$0.379 \pm 0.060$	$0.801 \pm 0.055$	$0.691 \pm 0.034$
Batch size = 32	$0.689 \pm 0.032$	$0.500 \pm 0.069$	$0.759 \pm 0.048$	$0.692 \pm 0.027$
W/o anti-aliasing	$0.708 \pm 0.017$	$0.527 \pm 0.066$	$0.767 \pm 0.051$	$0.708 \pm 0.024$
W/o discount	$0.712 \pm 0.026$	$0.462 \pm 0.032$	$0.825 \pm 0.037$	$0.731 \pm 0.023$
Full method	${0.725 \pm 0.025 }$	${0.448 \pm 0.063}$	${0.850 \pm 0.032}$	${0.746 \pm 0.016}$

**Table 2:** Comparing our full method to methods which omit one technique: ten runs best validation

Method variant	ROC–AUC	Sensitivity	Specificity	Accuracy
Baseline	0.639	0.613	0.562	0.575
W/o batch z-score	0.667	0.202	0.886	0.708
W/o $L_2$	0.680	0.539	0.794	0.727
Batch size = 64	0.696	0.488	0.759	0.689
Batch size = 32	0.725	0.636	0.667	0.659
W/o anti-aliasing	0.728	0.505	0.774	0.704
W/o discount	0.749	0.464	0.832	0.736
Full method	0.768	0.486	0.884	0.781

**Table 3:** Full method additional training runs: maximum ROC–AUC

Method variant	ROC–AUC	Sensitivity	Specificity	Accuracy
Full method	0.772	0.606	0.828	0.770

## Discussion

### Average accuracy

Our full model had the highest average ROC–AUC and highest and most consistent accuracy out of each of our variants. In our variant which omitted batch-wise *z*-scoring, we observe a significant increase in metric variance as well as the lowest average sensitivity and ROC–AUC. We hypothesize there is not enough variance in scale and bias in the training set without this augmentation. The variant without $L_2$ regularization struggled with ROC–AUC and specificity, while having slightly higher average sensitivity than our full method. Even considering the fact that our model only has $\approx$ 165 K trainable parameters, without $L_2$ kernel regularization there is clear evidence that a small number of features are overemphasized. Our variant without anti aliasing has a higher sensitivity than our full method. However, this comes at a significant cost in specificity. We hypothesize that this is due to the model associating aliasing with the presence of the onset, and that anti-aliasing and/or removal of high frequency information is important for reducing the frequency of false positives. The variant without loss discounting was the closest to our best results, trading off more specificity than was gained in sensitivity. In both cases, increasing the batch size from 16 has a significant negative impact on ROC–AUC during validation. Our baseline model predictably had the worst results overall.

### Maximum accuracy

We observe our full method has highest single epoch ROC–AUC of each variant. All of our variants appear to be heavily dependent on weight initialization and mini-batch batch selection during training, with many separate training runs needed to achieve highest generalization. We hypothesize that this is due to both the paucity of the data set and unstable gradients caused by lower batch sizes.

In addition to the ten runs for our full method, we conducted approximately twenty additional runs for our full method with new random seeds. In Table 3, We show the best overall model in terms of ROC-AUC. The model has much higher sensitivity without sacrificing a significant amount of specificity. We use this model for all following discussion and visualization of model behavior.

### Explaining model predictions

*Salience* In order to help explain our models predictions, we computed the gradient of *y* with respect to input sequences from the test set and summed the absolute value of the gradient for each feature channel together:

$$\begin{aligned} {\textit{salience}}(t) = \sum _{f=0}^{9} \left| \frac{\partial {\textit{y}}}{\partial {\textit{seq}}_{t,f}}\right| \end{aligned}$$

For visualization purposes, we normalize salience with the timestep containing the maximum value: ${\textit{salience}}_{vis}(t) = \frac{salience(t)}{salience(t_{max})}$. In each visualization we see only strong, sparse activation contributing to the models decision due to the GMP layer at the top of the network.

**Fig. 1:**
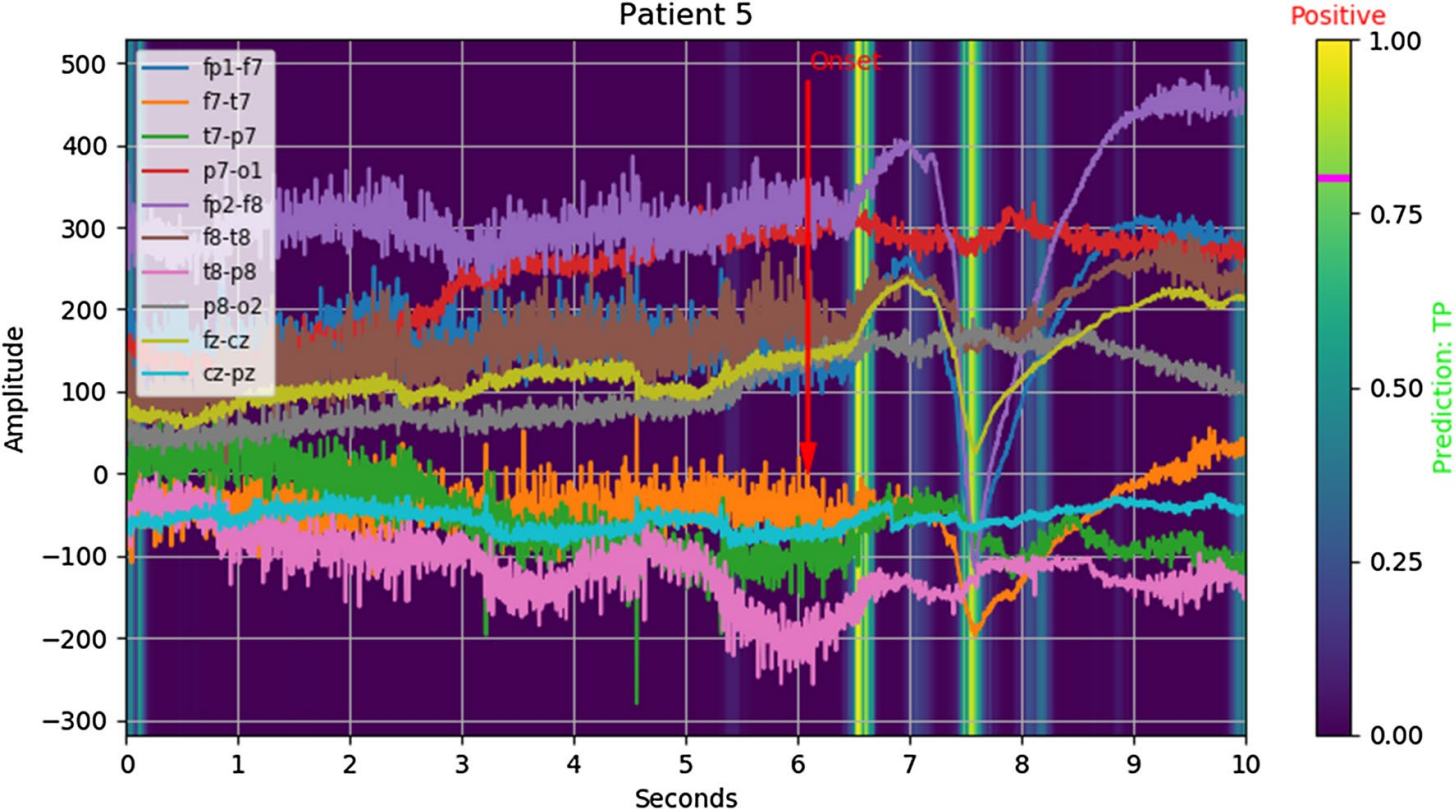
Salience: $y_{true} = 1, \lfloor y_{pred} \rceil = 1$ (true positive)

*Example: true positive* Arguably the strongest activation overall appears to happen when almost every channel simultaneously increases, which can happen several times around the onset. We visualize this in Fig. 1, where observe strong activation on the rising edge of a global increase.

**Fig. 2:**
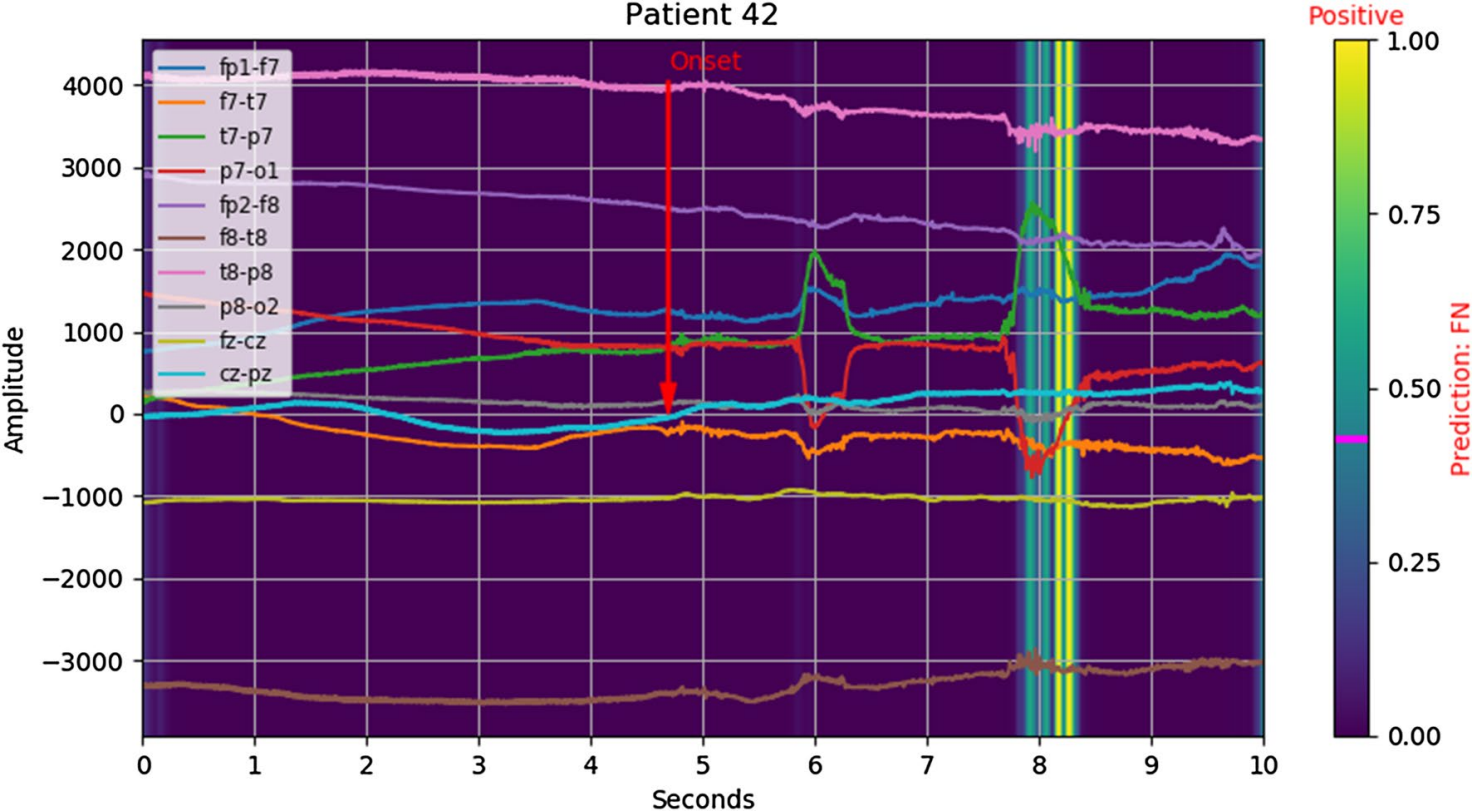
Salience: $y_{true} = 1, \lfloor y_{pred} \rceil = 0$ (false negative)

*Example: false negative* Only some instances of the onset of slow activity exhibit strong cross channel correlation, as demonstrated in Fig. 2. While most channels appear to move simultaneously, there is less positive correlation as well as some negative correlation between channels. In this particular example, there appears to be a wide spread of channel bias and low dynamic range. We hypothesis that *z*-scoring using the population mean and standard deviation may not be optimal for all examples, and that an adaptive strategy could improve validation performance.

**Fig. 3:**
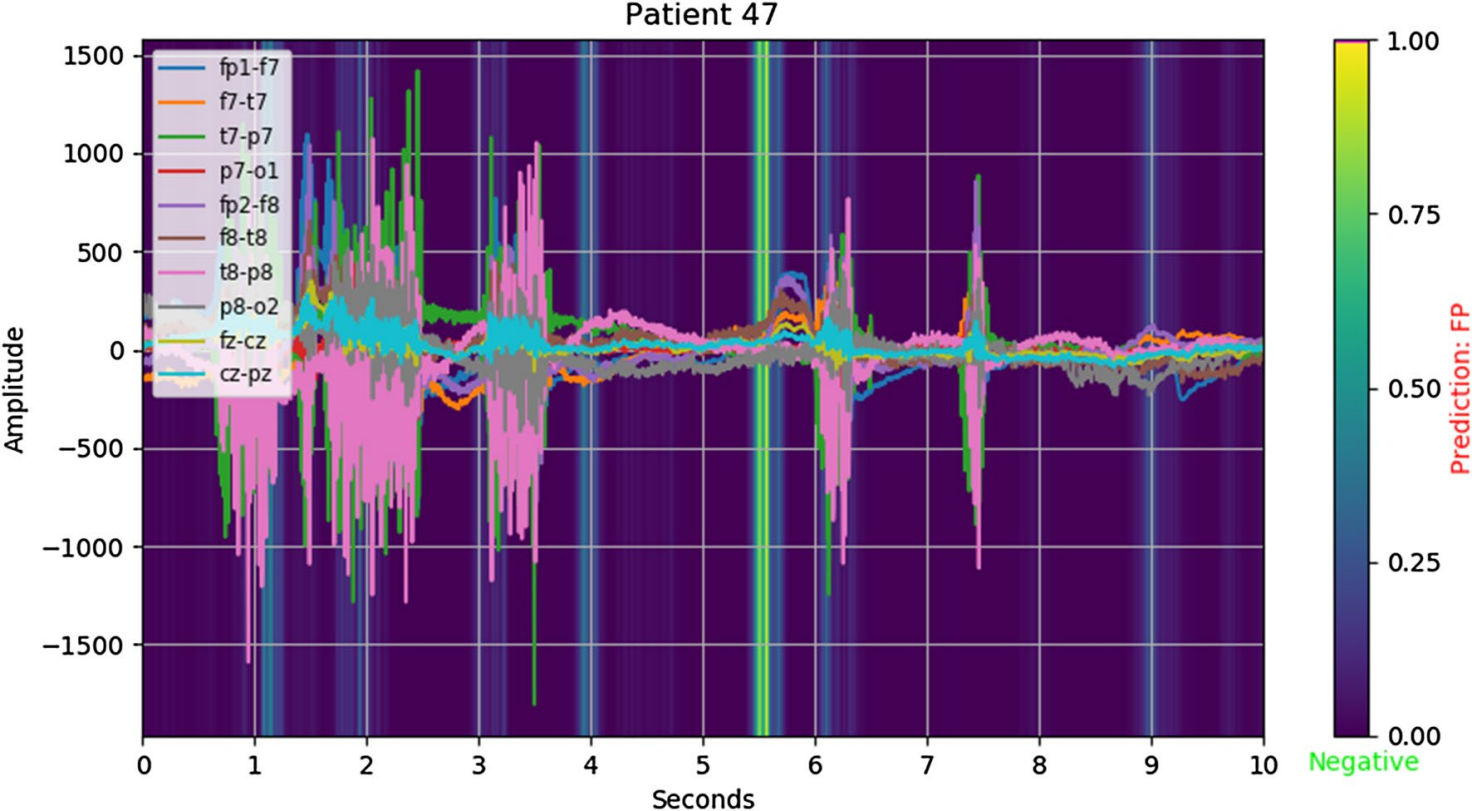
Salience: $y_{true} = 0, \lfloor y_{pred} \rceil = 1$ (false positive)

*Example: false positive* Fig. 3 demonstrates that not all instances of cross channel correlation are useful for predicting the onset by themselves. We hypothesize that a model may need to take into account the temporal nature of the problem in order to avoid these types of false positives.

**Fig. 4:**
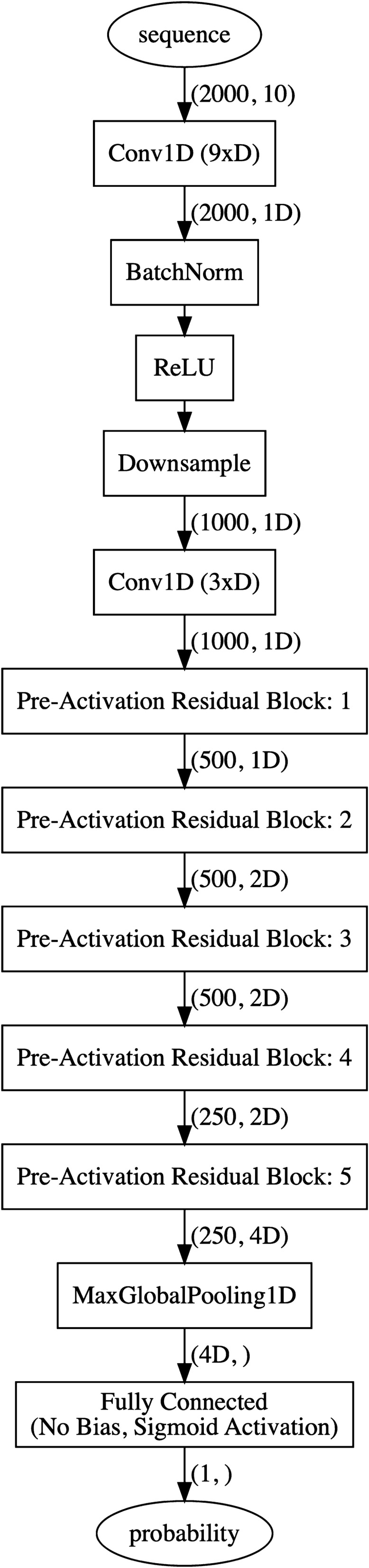
Our network has 12 convolutional layers, each of which is followed by batch normalization and a rectified linear unit. Residual connections are used to improve gradient propagation throughout the network

## Conclusions

While our naive baseline model had relatively poor accuracy, we demonstrated the impact of many different regularization techniques. It follows that deep learning can be an effective tool for signal detection problems with a small amount of available training data. By conducting our experiment over many different training runs, we show the statistical significance of our results. Finally, we demonstrated that while our model may be a black box, we can make the results easier to interpret with salience and effective visualization.

### Future work

We recognize that the loss discount factor could be made into a continuous function across the entire sequence. Currently, examples with a negative label could contain the start of the onset due to the the labeling task being particularly challenging, but are weighted as heavily as non ambiguous examples. In addition, we observed examples of false positives which would be relatively easy for a human to classify correctly due to drastic changes in overall behavior patterns. An improved model would be able to recognize these changes over time in addition to identifying channel cross correlation.

## Data Availability

The data include protected health information, thus are not publicly available.

## Abbreviations

EEG: Electroencephalogram
GAP: Global average pooling
GMP: Global max pooling
ROC-AUC: Receiver operator characteristic area under curve
SGD: Stochastic gradient descent
SUDEP: Sudden death in epilepsy
